# Single-fraction SRS and multiple-fraction SRT for brain metastases from colorectal cancer

**DOI:** 10.3389/fonc.2022.1060570

**Published:** 2022-12-06

**Authors:** Yong Li, Junlan Wu, Fenghua Liu, Xianjun Shao, Xiaohua Liang, Feifei Zhang, Yan Meng, Meihua Shen, Mianshun Pan

**Affiliations:** ^1^ Department of Radiation Oncology, Third Affiliated Hospital of Naval Medical University, Shanghai, China; ^2^ Center of Radiation Oncology, Chinese People's Armed Police Force Shanghai Corps Hospital, Shanghai, China; ^3^ Department of Oncology, Huashan Hospital Affiliated to Fudan University, Shanghai, China

**Keywords:** brain metastases, colorectal cancer, stereotactic radiotherapy, stereotactic radiosurgery, bevacizumab

## Abstract

**Objective:**

Brain metastasis from colorectal cancer (CRC) is rare. Although stereotactic radiotherapy (SRT) and stereotactic radiosurgery (SRS) are effective treatments for brain metastasis, reports on brain metastasis of CRC are limited. This study compared the efficacy of SRT and SRS for the treatment of brain metastases from CRC and analysed the related factors to reveal the specificity CRC-derived brain metastasis.

**Methods:**

A retrospective analysis of 116 patients with brain metastases from colorectal cancer was performed and included 56 patients in the SRT group and 60 patients in the SRS group. The clinical characteristics of the two groups were analysed, and the local tumour control rate, overall survival time and radiation-induced brain injury were compared between the two groups.

**Results:**

The objective response rates of the SRT and SRS groups were 76.8% and 66.7%, respectively, while the local control rates at 6 months were 87.5% and 81.6%, respectively, and no significant differences were observed between the groups (P=0.295). The median overall survival time was 10.3 months for all patients and was 10.9 months in the SRT group and 9.8 months in the SRS group, with no significant difference between the groups (P=0.123). A multivariate analysis showed that the main factors of poor prognosis were low GPA score (P=0.002), KRAS mutation (P=0.035), extracranial metastasis (P=0.005) and no bevacizumab treatment (P=0.001). No significant difference was observed in the incidence of acute and late radiation-induced injury between the two groups.

**Conclusion:**

Both SRT and SRS are effective methods for the treatment of CRC-derived brain metastases. The simultaneous use of bevacizumab may be one of the most important factors that affects the survival of these patients.

## Introduction

Brain metastasis from colorectal cancer (CRC) is rare, and according to one study that analysed 541,244 CRC patients, the incidence of brain metastasis was only 2.1% (1). Other studies have also reported that the incidence of brain metastasis in Asian CRC patients may be as high as 7% (2). CRC is one of the most common tumours worldwide, and the survival time of these patients has gradually improved with advancements in systemic therapy. Brain metastasis from CRC is also becoming increasingly common (3).

Stereotactic radiosurgery (SRS) is an important treatment modality for brain metastases, as the one-year tumour control rate ranges from 62.4% to 92.9% (4, 5). With the extensive application of SRS, these patients experience longer survival. Radiation-induced brain necrosis that was historically rare has become more common, which has seriously affected the quality of life of these patients and even leads to death (6). Radiobiological studies have confirmed that multiple-fraction radiotherapy may be beneficial, as this method kills tumour cells in different stages of the cell cycle and protects normal brain tissue (7). The present study included multicentre data to analyse the efficacy of multifractionated SRT and single-fraction SRS in the treatment of CRC brain metastases and analysed the relevant factors.

## Materials and methods

### Patients and diagnoses

The patients in this study were from the Chinese brain metastasis study group database, which includes data from 36 hospitals in China. The inclusion criteria for the patients in this study were as follows: (I) the patients received SRS or SRT at least once, (II) the pathological diagnosis of all the primary colorectal lesions was adenocarcinoma, and (III) the patients had complete follow-up with enhanced magnetic resonance imaging (MRI) data available. We excluded patients younger than 18 years of age and those with severe liver or kidney dysfunction. In all, 116 cases were included in this study: 56 cases in the SRT group and 60 cases in the SRS group.

### SRS and SRT

Multiple-fraction SRT was administered by a gamma ray stereotactic radiotherapy system (SGS-I, Huiheng, China) or a Cyberknife^®^ system (Accuracy Inc., Sunnyvale, CA, USA). The head and neck were fixed by a thermoforming mask and then scanned with enhanced computed tomography (CT). The gross tumour volume (GTV) was outlined on the enhanced CT image, and some patients underwent image fusion with enhanced MRI. The planning target volume (PTV) is formed by expanding the GTV by 1-3 mm. The prescription dose is 3-10 Gy/fraction over 3-10 fractions, and the total dose is 30-45 Gy. The biological effective dose (BED) in different dose fractions was evaluated using the LQ formula: BED = nd [1+d/(α/β)],α/β=10. The BED of SRT was 50-72 Gy (median 60.5). The single-fraction SRS was performed with various gamma knife models. The stereotactic head frame was fixed under local anaesthesia and then scanned using enhanced MRI. The PTV was formed by expanding the GTV by 1-3 mm. The prescription dose was 16-22 Gy, and the BED was 41.6-70.4 Gy (median 59.5). If patients exhibited new or recurrent intracranial lesions during follow-up, SRT, SRS, whole-brain radiotherapy (WBRT) and/or other systemic treatment was used as the rescue treatment.

### Follow-up and evaluation

Follow-up was performed every 1-3 months, and tumour responses were evaluated on enhanced MRI according to the RECIST 1.1 criteria (1). Complete response (CR): All target lesions disappeared completely (2). Partial response (PR): The total diameter of the target lesion was reduced by ≥30% (3). Progressive disease (PD): The total diameter of the target lesion increased by ≥20% (4). Stable disease (SD): the degree of reduction of the target lesion did not reach PR or the degree of increase did not reach PD. The ratio of CR+PR is defined as the objective response rate (ORR), while the ratio of CR+PR+SD is defined as the local control rate (LCR). Radiation-induced brain necrosis was diagnosed when at least 2 of the criteria listed below were fulfilled (8) (1): On enhanced MRI or CT images, the lesions showed irregular borderP enhancement with obvious oedema (2); MRI or CT perfusion imaging showed hypoperfusion in the lesion (3); MRS suggested a decreased Cho value in the enhanced area (4); PET revealed that the uptake of ^18^F-FDG in the enhanced area was significantly decreased. Overall survival (OS) was defined as the time from the diagnosis of brain metastasis to death or the end of the follow-up period.

**Statistical analysis**

Data are presented as the mean ± the standard deviation. The variables were compared using an independent sample t test. Categorical variables were compared using the chi-squared test. The local control time and overall survival were analysed by the Kaplan−Meier actuarial method, with statistical significance determined by the log-rank test. Cox regression analysis by the forward stepwise method was used to verify the independent variables of all potential predictors. Statistical analysis was performed with SPSS 23.0 software (SPSS, Chicago, IL). A P value < 0.05 was considered significant.

## Results

### Baseline characteristics

The characteristics of all 116 patients with CRC brain metastases are shown in **Table 1**. In all, 66.4% of the patients were younger than 60 years, and 81.0% of the patients had 1-3 lesions. The proportion of patients with KRAS mutations was 71.6%, and 86.2% of the brain metastases originated from the left colon and rectum. Overall, 54.3% of patients had liver metastasis, 78.4% had lung metastasis, and 43.9% had multiorgan metastasis. Due to the late disease stage, all patients received systemic treatment, including chemotherapy and some targeted therapy, and 57.8% of the patients were treated with bevacizumab. The GTV in the SRS group was smaller than that in the SRT group (P=0.003). No significant differences were observed in other characteristics between the two groups.

**Table 1:** Summary characteristics of patients in the SRT and SRS groups.

Variables	Total (n=116)	SRT group (n=56)	SRS group (n=60)	P value
Age (y)				
< 60	77 (66.4%)	35 (62.5%)	42 (70.0%)	0.393
≥ 60	39 (33.6%)	21 (37.5%)	18 (30.0%)	
Sex				
Male	51 (44.0%)	24 (42.9%)	27 (45.0%)	0.816
Female	65 (56.0%)	32 (57.1%)	33 (55.0%)	
Number of lesions				
1~3	94 (81.0%)	44 (78.6%)	50 (83.3%)	0.513
> 3	22 (19.0%)	12 (21.4%)	10 (16.7%)	
GTV (cm^3^)				
< 4	54 (46.6%)	17 (30.4%)	37 (61.7%)	0.003
4~14	49 (42.2%)	31 (55.4%)	18 (30.0%)	
> 14	13 (11.2%)	8 (14.3%)	5 (8.3%)	
GPA score				
≤1.0	16 (13.8%)	10 (17.9%)	6 (10.0%)	0.147
1.5~2.5	57 (49.1%)	30 (53.6%)	27 (45.0%)	
≥3.0	43 (37.1%)	16 (28.6%)	27 (45.0%)	
Gene mutation				
KRS mutation	83 (71.6%)	42 (75.0%)	41 (68.3%)	0.426
Others	33 (28.4%)	14 (25.0%)	19 (31.7%)	
Target BED (Gy)				
≥ 60	93 (80.2%)	44 (78.6%)	49 (81.7%)	0.676
< 60	23 (19.8%)	12 (21.4%)	11 (18.3%)	
Location of primary tumor				
Right colon	16 (13.8%)	6 (10.7%)	10 (16.7%)	0.353
Left colon and rectum	100 (86.2%)	50 (89.3%)	50 (83.3%)	
Extracranial metastasis				
Liver	63 (54.3%)	30 (53.6%)	33 (55.0%)	0.235
Lung	91 (78.4%)	41 (73.2%)	50 (83.3%)	
Multi-organ	51 (43.9%)	22 (39.3%)	29 (48.3%)	
Bevacuzumab				
Used	67 (57.8%)	35 (62.5%)	32 (53.3%)	0.318
No	49 (42.2%)	21 (37.5%)	28 (46.7%)	

### Control of brain metastases

**Figure 1:**
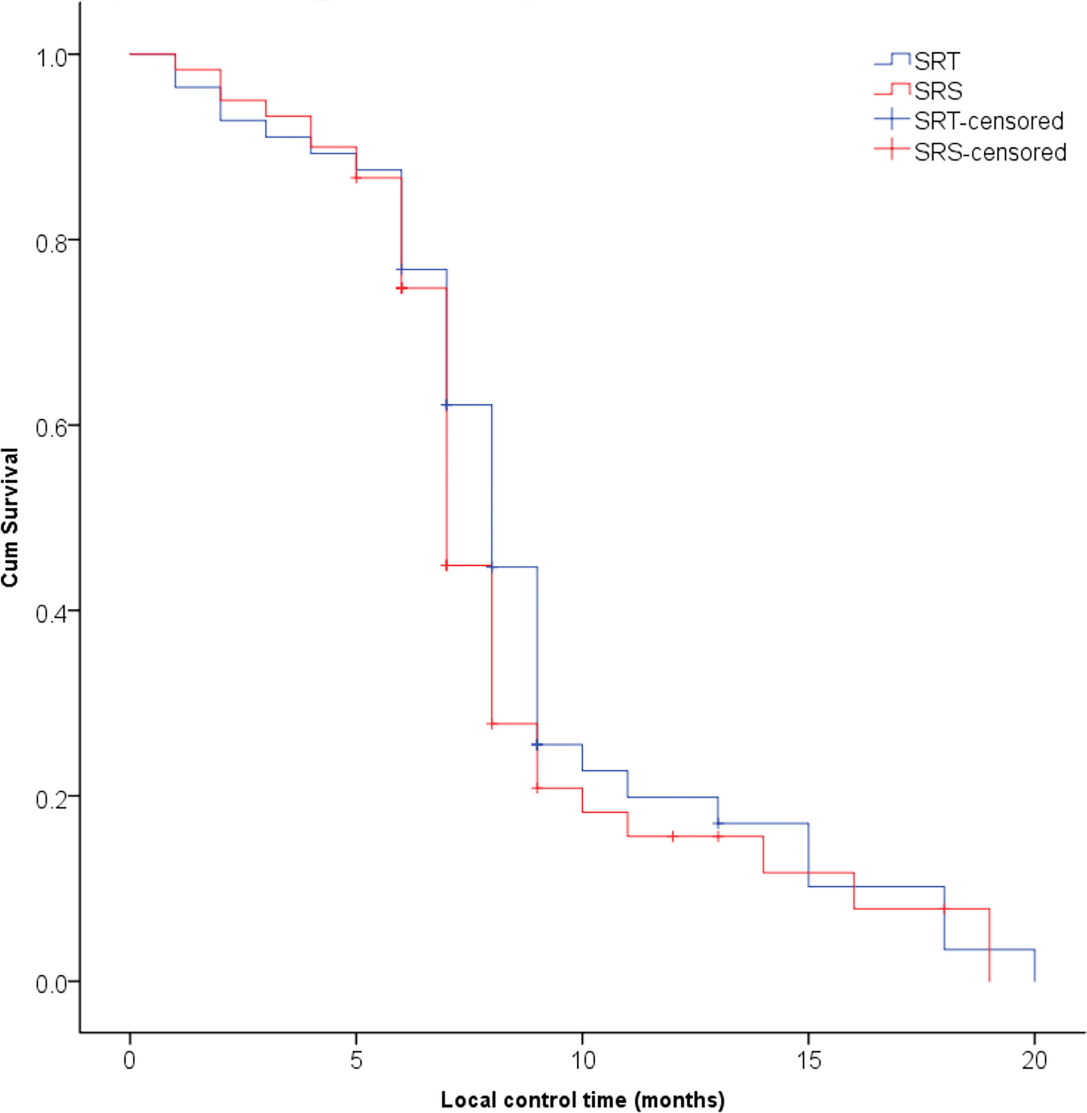
No significant difference was observed in the local control rate of brain metastases between the SRT and SRS groups (P = 0.295).

The median follow-up time of these patients was 10.7 months (range 5.6-38.0 months). The proportions of patients who achieved CR, PR, SD and PD in the SRT and SRS groups were 17.9% (10/56) and 13.3% (8/60), 58.9% (33/56) and 53.3% (32/60), 10.7% (6/56) and 15.0% (9/60), and 12.5% (7/56) and 18.3% (11/60), respectively. The ORRs of the SRT and SRS groups were 76.8% (43/56) and 66.7% (40/60), respectively, and no significant difference was observed between the two groups (P=0.662). The 3-month LCRs of the SRT and SRS groups were 92.9% (52/56) and 93.3% (56/60), respectively, while the 6-month LCRs were 87.5% (49/56) and 81.6% (49/60), respectively, and the differences between the groups were not significant (P=0.295, **Figure 1**). The distant intracranial failure rate was 53.6% (30/56) in the SRT group and 45.0% (27/60) in the SRS group. The rate of repeated use of SRT or SRS as salvage treatment was 80.0% (24/30) in the SRT group and 88.9% (24/27) in the SRS group.

### Survival analysis

**Figure 2:**
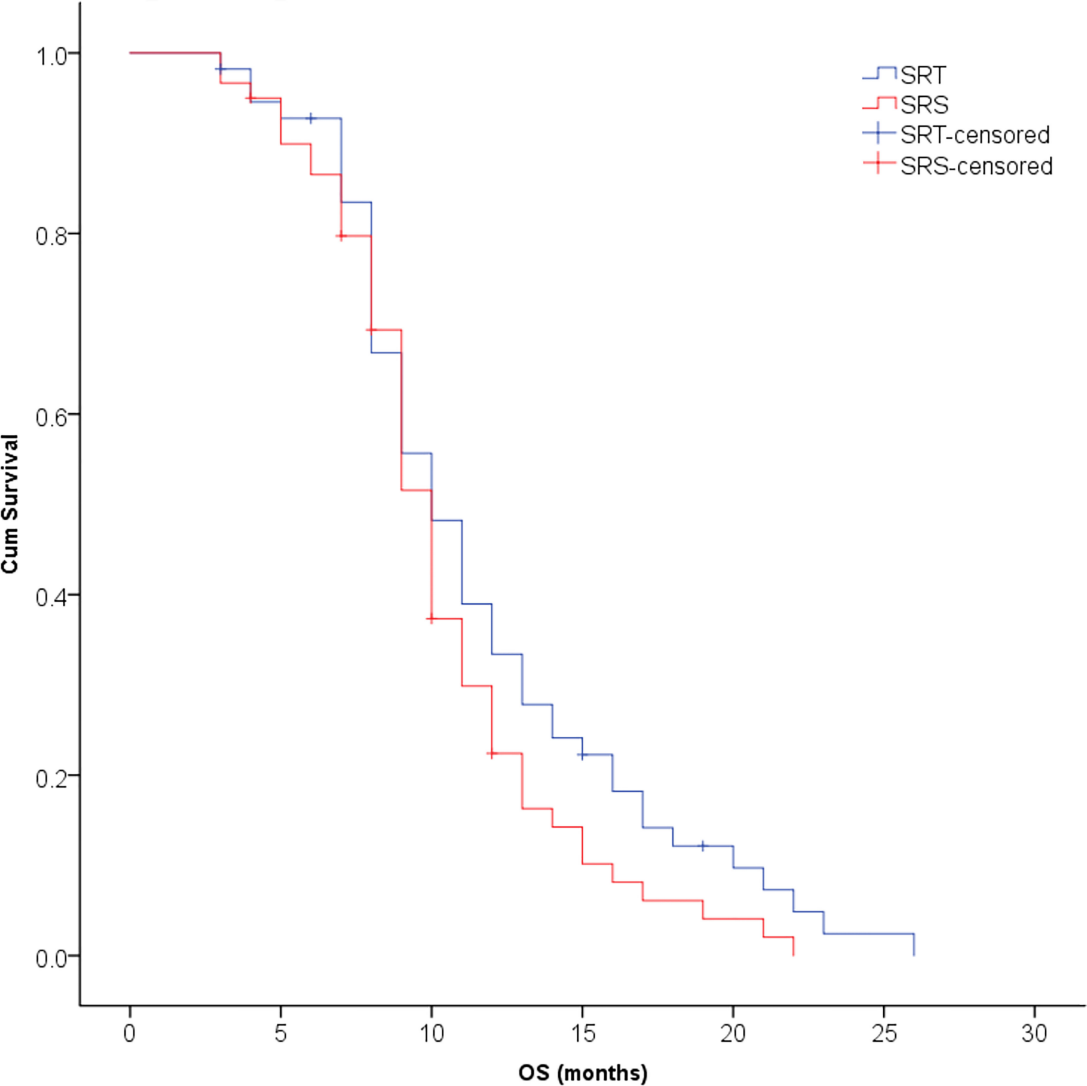
No significant difference was observed in overall survival between the SRT and SRS groups (P = 0.123).

**Figure 3:**
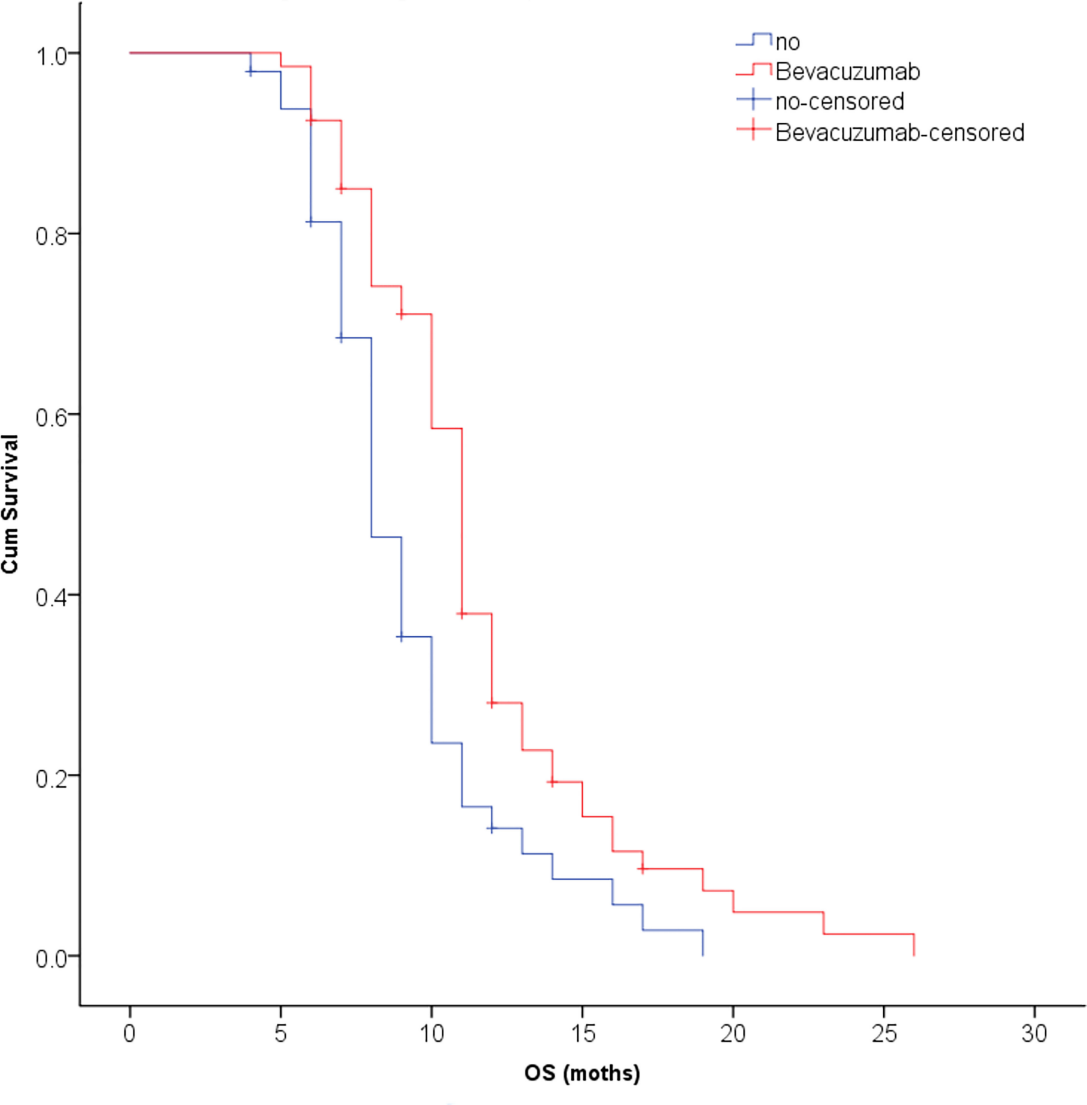
The multivariate analysis showed that bevacizumab could significantly improve the survival time of patients with CRC brain metastases (P = 0.001).

The median survival time was 10.3 months for all patients and was 10.9 months in the SRT group and 9.8 months in the SRS group; this difference was not significant (P=0.123, **Figure 2**). The univariate analysis showed that patients with 1 to 3 lesions (P=0.032), high GPA score (P=0.003), no KRAS gene mutation (P=0.001), and fewer extracranial metastases (P=0.036) as well as those who were treated with bevacizumab (P=0.001) had a longer overall survival (**Table 2**). The multivariate analysis showed that the main factors of poor prognosis were (**Table 3**) low GPA score (P=0.002), KRAS mutation (P=0.035), extracranial metastasis (P=0.005) and no use of bevacizumab (P=0.001, **Figure 3**).

**Table 2:** Univariate analysis of variables predictive of OS.

Variables	N	OS (m)	P value
Sex			
Male	51	10.2 ± 3.17	0.265
Female	65	8.9 ± 2.23	
Age (y)			
<60	77	10.5 ± 3.52	0.232
≥60	39	9.1 ± 3.29	
Number of lesions			
1-3	94	10.9 ± 3.55	0.032
> 3	22	8.8 ± 2.16	
GTV(cm3)			
< 4	54	10.3 ± 4.66	0.132
4~14	49	9.2 ± 2.17	
> 14	13	8.9 ± 2.38	
GPA score			
≤1.0	16	7.2 ± 1.81	0.003
1.5-2.5	57	10.8 ± 2.60	
≥3.0	43	14.1 ± 5.32	
Gene mutation			
KRS mutation	83	7.9 ± 3.08	0.001
No	33	11.8 ± 2.26	
Target BED(Gy)			
≥ 60	93	11.2 ± 3.26	0.105
< 60	23	9.1 ± 1.28	
Treatment			
SRT	56	10.9 ± 2.88	0.123
SRS	60	9.8 ± 3.15	
Location of primary tumor			
Right colon	16	9.1 ± 1.08	0.081
Left colon and rectum	100	10.2 ± 3.35	
Extracranial metastasis			
Liver	63	9.3 ± 2.61	0.036
Lung	91	10.2 ± 2.17	
Multi-organ	51	7.7 ± 2.38	
Bevacuzumab			
Used	67	11.7 ± 2.38	0.001
No	49	8.3 ± 2.26	

**Table 3:** Multivariate analysis of variables predictive of OS.

Variables	HR	95% CI	P value
Number of lesions	1.213	0.722-1.758	0.126
GPA score	0.523	0.519-1.126	0.002
KRAS mutation	1.713	1.116-2.332	0.035
Extracranial metastasis	1.557	1.122-2.778	0.005
Bevacuzumab	1.889	1.178-2.386	0.001

### Complications and toxicity

**Figure 4:**
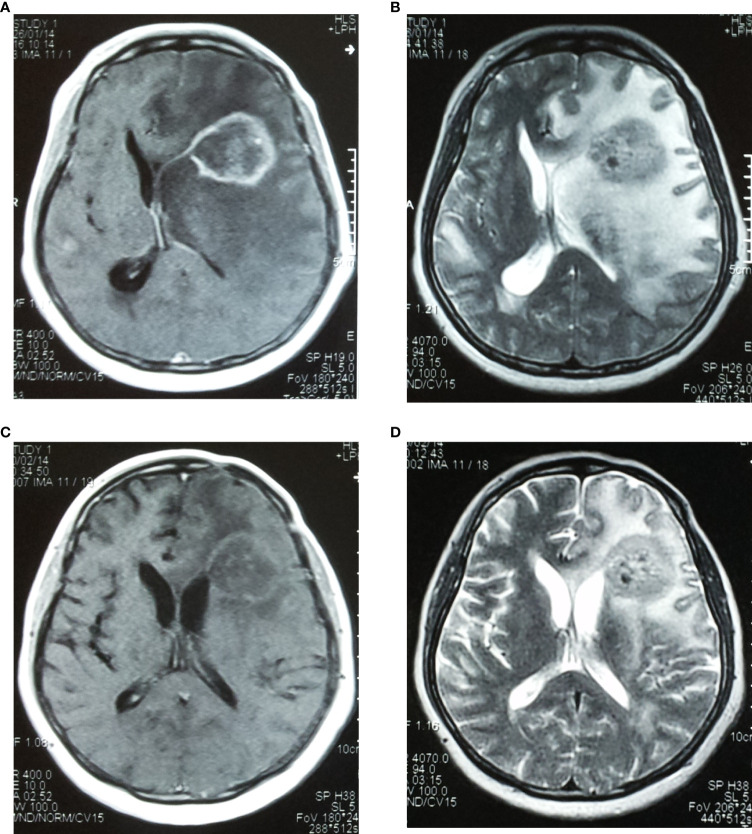
A case of brain metastasis derived from colon cancer with radiation-induced brain necrosis in the left frontal lobe that was then treated with 5 mg/kg bevacizumab. **(A)** Before treatment, MRI T1 showed enhanced areas of brain necrosis; **(B)** Before treatment, MRI T2 showed obvious perifocal oedema. **(C, D)** Two weeks after treatment, the area of oedema was obviously decreased, and the quality of life of the patient was significantly improved.

Steroids or glucocorticoids were routinely given to patients with neurological deficits or intracranial hypertension caused by brain metastases. No radiotherapy-induced epilepsy was found during radiotherapy or one month after radiotherapy. The rates of acute radiation-induced reactions, including headache, nausea and vomiting as well as aggravation of the original neurological dysfunction, in the SRT and SRS groups were 8.9% (5/56) and 16.7% (10/60), respectively, with no significant difference between the groups (P=0.215). The incidence of late radiation-induced brain necrosis in the SRT and SRS groups was 12.5% (7/56) and 21.7% (13/60), respectively, with no significant difference between the groups (P=0.192). These patients improved after supportive treatment, and some patients with refractory symptoms experienced significant relief after treatment with bevacizumab (**Figure 4**).

## Discussion

The presence of brain metastasis indicates late-stage CRC, as 36.6–68% of patients have liver metastasis and 71–92% of patients have lung involvement (9). The median survival of these patients after symptomatic treatment is only 4-6 weeks, whereas their survival after systemic chemotherapy is approximately 6-9 months (10, 11). The local treatment of brain metastases primarily includes surgery, SRS and WBRT. One study showed that in patients with solitary brain metastases from non-small cell lung cancer, the median survival time was 12.7 months in surgery group and 14.85 months in SRS group, respectively. The 1-year, 2-year and 5-year survival rates after surgery vs. SRS were 59% vs. 62%, 33% vs. 33% and 19% vs. 14%, respectively. However, no significant difference was observed between the two treatments (12). The results of some studies have shown that SRS alone results in a similar overall survival compared with SRS+WBRT for a limited number and volume of brain metastases (13, 14). Surgical resection of brain metastases from CRC in selected patients may help prolong survival. Additional SRS or WBRT following surgery is valuable in improving prognosis (2, 15). Therefore, SRS has become the main treatment for brain metastases, while WBRT is only used for palliative treatment of patients with multiple brain metastases and in those with meningeal involvement.

Increasing the radiation dose may improve the tumour control rate, but this will inevitably lead to radiation-induced brain injury. In clinical practice, for tumours with large volumes and those in special anatomical sites, it is necessary to reduce the single radiation dose or increase the number of radiation fractions to ensure treatment safety. Radiobiological studies have demonstrated that multi-fraction irradiation may induce sensitivity in tumour cells that are resistant. Multi-fraction irradiation can also increase the probability of reoxygenation of hypoxic tumour cells, which thus increases the sensitivity of tumour cells to radiation. Multi-faction radiation can not only kill tumour cells to the maximum extent but can also better protect brain tissue that undergoes delayed-reaction injury. A meta-analysis showed that for metastatic brain tumours with a diameter of 2-3 cm, the 1-year control rates of single and fractional radiosurgery were 77.6% and 92.9%, respectively (P=0.18), while the incidence of brain necrosis was 23.1% and 7.3%, respectively (P=0.003). Compared with traditional single SRS, fractional SRT may reduce the incidence of radiation-induced brain necrosis and simultaneously result in a similar or higher tumour control rate (5). This study showed that the ORR of the SRT group was slightly higher than that of the SRS group. The incidence of acute radiation reactions and radiation-induced brain necrosis in the SRT group was only half of that in the SRS group. This may be due to the small number of cases, and no significant difference was found between the two groups. Interestingly, the GTV volume of the SRS group was smaller than that of the SRT group, but the tumour control rate and the incidence of brain injury were similar, which indicates that fractional radiotherapy is advantageous for large tumours.

This study showed that most of the CRC brain metastases were limited in number, and 82.7% of the patients had 1 to 3 lesions, which was suitable for SRT/SRS treatment. Although brain metastasis occurs in late-stage tumours, if these intracranial lesions can be eliminated or controlled as soon as possible and are also sensitive to systemic treatment, including chemotherapy and targeted therapy, survival time may be improved. Bevacizumab can treat CRC-derived brain metastasis effectively, and this therapy also improves the symptoms of radiation-induced brain necrosis. Bevacizumab can significantly improve the quality of life and prolong the survival of these patients. The multivariate analysis in this study showed that, with timely SRS/SRT and bevacizumab treatment, the number of lesions may not be the primary determinant of survival.

Studies have shown that up to 96.8% of CRC patients with brain metastases exhibit no obvious symptoms related to brain metastases at the time of initial diagnosis (1). Therefore, according to the patient characteristics in this study, for patients with primary rectal lesions, young patients, and those with lung metastases, more metastatic sites, and KRAS gene mutations, it is recommended that regular enhanced MRI examinations of the brain be performed to detect and treat these lesions earlier.

Many different genes may be mutated during the occurrence and development of CRC brain metastases. One study conducted an analysis of whole-exome sequencing and whole-genome sequencing data on 19 trios of patient-matched brain metastases, primary CRC tumours, and adjacent normal tissue. Compared with primary CRC, brain metastases exhibited elevated mutational signatures of homologous recombination deficiency and mismatch repair deficiency. Further analysis revealed that two DNA damage response signatures could emerge early and were enhanced in brain metastasis tissues but were absent in matched primary CRC tissues. Brain metastasis-specific mutations in DNA damage response genes and elevated microsatellite instability levels support the importance of the DNA damage response in brain metastasis of CRC. These findings reveal the vast genomic difference between metastatic and primary tumours and suggest that PARP inhibitors and anti-PD-1 drugs may have clinical potential to prevent and treat DNA damage response-deficient brain metastases (16). A higher number of KRAS mutations were observed in CRC brain metastatic tumours than in primary tumours (56% vs. 74%) (17). Similarly, the proportion of patients with KRAS mutations in this study was 72.4%. These mutant clone populations may become highly invasive, break through the blood−brain barrier, and finally form brain metastases. The multivariate analysis showed that KRAS mutations may indicate short survival. Therefore, the diagnosis, treatment and prevention of CRC brain metastases may require more in-depth research at a molecular mechanistic level.

In conclusion, the incidence of CRC brain metastasis is low, but with the progress in treatment methods and the prolongation of patient survival, brain metastasis of CRC is becoming increasingly common. Since this is a retrospective study with a small number of cases, selection bias may have occurred. Our results suggest that both SRT and SRS are effective methods for the treatment of CRC brain metastases. The simultaneous use of SRT or SRS and systemic therapy represented by bevacizumab may be one of the most important factors that affects the survival of these patients. These findings may provide a reference for future prospective studies.

## Data availability statement

The original contributions presented in the study are included in the article/supplementary material. Further inquiries can be directed to the corresponding authors.

## Ethics statement

The studies involving human participants were reviewed and approved by ethics committees of Shanghai Wujing Hospital (WJE-01) and Third Affiliated Hospital of Naval Medical University (20220610). The patients/participants provided their written informed consent to participate in this study. Written informed consent was obtained from the individual(s) for the publication of any potentially identifiable images or data included in this article.

## Author contributions

MP, MS, YM and YL set up the study design and study protocols. YL, JW, FL, XS, XL and FZ participated in performing the research. YL drafted the manuscript. All authors contributed to the article and approved the submitted version.

## Funding

The National Key R&D Program of China (2022YFC2503700, 2022YFC2503704); Application demonstration of colorectal cancer intelligent diagnosis and treatment system (20DZ1100106).

## Conflict of interest

The authors declare that the research was conducted in the absence of any commercial or financial relationships that could be construed as a potential conflict of interest.

## Publisher’s note

All claims expressed in this article are solely those of the authors and do not necessarily represent those of their affiliated organizations, or those of the publisher, the editors and the reviewers. Any product that may be evaluated in this article, or claim that may be made by its manufacturer, is not guaranteed or endorsed by the publisher.
